# A Population Dynamics Model for Clonal Diversity in a Germinal Center

**DOI:** 10.3389/fmicb.2017.01693

**Published:** 2017-09-12

**Authors:** Assaf Amitai, Luka Mesin, Gabriel D. Victora, Mehran Kardar, Arup K. Chakraborty

**Affiliations:** ^1^Chemical Engineering, Massachusetts Institute of Technology Cambridge, MA, United States; ^2^Institute for Medical Engineering and Science, Massachusetts Institute of Technology Cambridge, MA, United States; ^3^Ragon Institute of MGH, MIT and Harvard Cambridge, MA, United States; ^4^Laboratory of Lymphocyte Dynamics, Rockefeller University New York, NY, United States; ^5^Physics, Massachusetts Institute of Technology Cambridge, MA, United States; ^6^Biological Engineering and Chemistry, Massachusetts Institute of Technology Cambridge, MA, United States

**Keywords:** germinal center reaction, population dynamics, modeling and simulations, clonal evolution, affinity maturation

## Abstract

Germinal centers (GCs) are micro-domains where B cells mature to develop high affinity antibodies. Inside a GC, B cells compete for antigen and T cell help, and the successful ones continue to evolve. New experimental results suggest that, under identical conditions, a wide spectrum of clonal diversity is observed in different GCs, and high affinity B cells are not always the ones selected. We use a birth, death and mutation model to study clonal competition in a GC over time. We find that, like all evolutionary processes, diversity loss is inherently stochastic. We study two selection mechanisms, birth-limited and death limited selection. While death limited selection maintains diversity and allows for slow clonal homogenization as affinity increases, birth limited selection results in more rapid takeover of successful clones. Finally, we qualitatively compare our model to experimental observations of clonal selection in mice.

## Introduction

Upon natural infection or vaccination, antibodies develop in domains within secondary lymphoid organs called germinal centers (GC), which appear shortly after infection (Victora and Nussenzweig, 2012). B cells with some threshold affinity for the antigen can seed GCs and, with help from several other types of immune cells, undergo affinity maturation (AM) (Eisen and Siskind, 1964), which is an evolutionary process of mutation, competition and proliferation, that ultimately generates high affinity antibodies.

At the initial stage of the GC reaction (GCR), naïve B cells are recruited. During AM, the AID protein induces random mutations in the gene coding for the BCR at a high rate (Muramatsu et al., 2000). A GC is not histologically uniform but divided roughly into two areas: a dark zone (DZ) and a light zone (LZ). After proliferating and mutating in the DZ, B cells migrate to the LZ, where they consume antigen displayed on the surface of follicular dendritic cells, and display antigen-derived peptide-MHC complexes on their surface. These B cells then compete for limiting amounts of T follicular helper cells (TfhCs). Following a proliferation signal from TfhCs (Rolf et al., 2010), the majority of B cells migrate back to the DZ, while a few differentiate in to antibody-producing plasma cells and memory cells (Oprea and Perelson, 1997). Iterative cycles of such hypermutation and selection result in both an increase in B cell affinity over time, and the loss of B cell clones in the competition process, such that a few successful clones are thought to remain at the end of the GCR (Jacob et al., 1993). After roughly 2 weeks, although this time can vary significantly, the process stops and the GC collapses.

The number of founding clones of a GC was traditionally thought to be between 1 and 6 (Kroese et al., 1987; Liu et al., 1991; Jacob et al., 1993). However, a recent study has shown that the initial number of clones is much higher, of the order of 50–200 initial clones, and that the clonal number variability after 3 weeks remains high (Tas et al., 2016). The experimental system uses the “brainbow” allele for multicolor fate mapping to permanently tag individual B cells and their progeny with different combinations of fluorescent proteins (Livet et al., 2007), resulting in up to 10 different colors. Thus, a number of distinct observable sub-clonal lineages emerge when a cell belonging to a certain clone chooses a color. The sub-clonal lineages are observed at different time points of the GCR (Tas et al., 2016). This method underestimates the number of clones in very diverse GCs (Tas et al., 2016) as not all clones choose a color, and multiple clones can choose the same color. Since recombination occurs after the initial clone has proliferated, multiple colors may represent the same clone. However, the method provides a high throughput estimate of GC clonality. Moreover, GC clonal diversity was also estimated by sequencing B cells, which allows for exact reconstruction of the lineages, and both methods point to the same qualitative behavior. Surprisingly, it was found that while clonal diversity is lost with time, the number of remaining clones varied significantly between GCs, even ones from the same lymph node that shared many clones.

AM has been modeled extensively over the last 30 years (Brink, 2007; Chan et al., 2013), dating back to the seminal work of Perelson et al., showing that cycling of B cells between the DZ and the LZ is optimal for affinity gain (Kepler and Perelson, 1993; Oprea and Perelson, 1997). Meyer-Hermann et al. (2012) developed very detailed simulations capbable of reproducing the dynamics and interactions of individual B and T cells within a GC. More recently, several computational studies (Chaudhury et al., 2014; Luo and Perelson, 2015; Wang et al., 2015; Shaffer et al., 2016) have investigated the effect of different immunization strategies with multiple variant antigens on the development of cross-reactive antibodies. Many of these models assume that selection is done by eliminating cells with low affinity BCR (Figge, 2005; Zhang and Shakhnovich, 2010). However, new evidence suggests that the extent of B cell proliferation in the DZ is proportional to the strength of the signal the B cell has received in the LZ (Victora et al., 2010; Gitlin et al., 2014, 2015) which can lead to rapid expansion of the progeny of a selected cell. We denote these two scenarios “death-limited” and “birth-limited” selection respectively. Since there is a minimum threshold for any response, and proliferation is related to BCR affinity, we suggest that both are needed to explain AM. We use here tools from population dynamics and stochastic processes to show that the AM process and clonal selection can be understood in terms of stochastic clonal competition, leading to an inherently probabilistic selection of fitter clones.

We estimate numerically clonal loss (*homogenization*) in a GC and show that the magnitude by which affinity changes per single mutation is the determinant factor in explaining clonal homogenization rate. Because clonal selection is a stochastic process, we show that clonal diversity has a large variability between different GCs. While we do not include spatial resolution of B cell LZ-DZ migration (Figge et al., 2008), recycling of antibodies (Zhang et al., 2013), the model captures qualitatively the essence of clonal selection with effective rates of birth, death and mutation. We suggest that the basic aspects of clonal diversity in the GC can be captured using simple population dynamics models.

## Model description

### AM as a birth-death-mutation process

We model B cell proliferation and death during the GCR using a birth-death (BD) process (Renshaw, 1991). AID mutates the gene encoding for the BCR (Muramatsu et al., 2000) and as a consequence, affinity for the antigen changes. The resulting increase (or decrease) in affinity translates to a higher (lower) fitness of the B cell. Regarding the stochastic variation of BCR in affinity space as a form of diffusion, the model resembles a “birth-death-diffusion process” (Adke and Moyal, 1963).

### Growth phase

**Figure 1:**
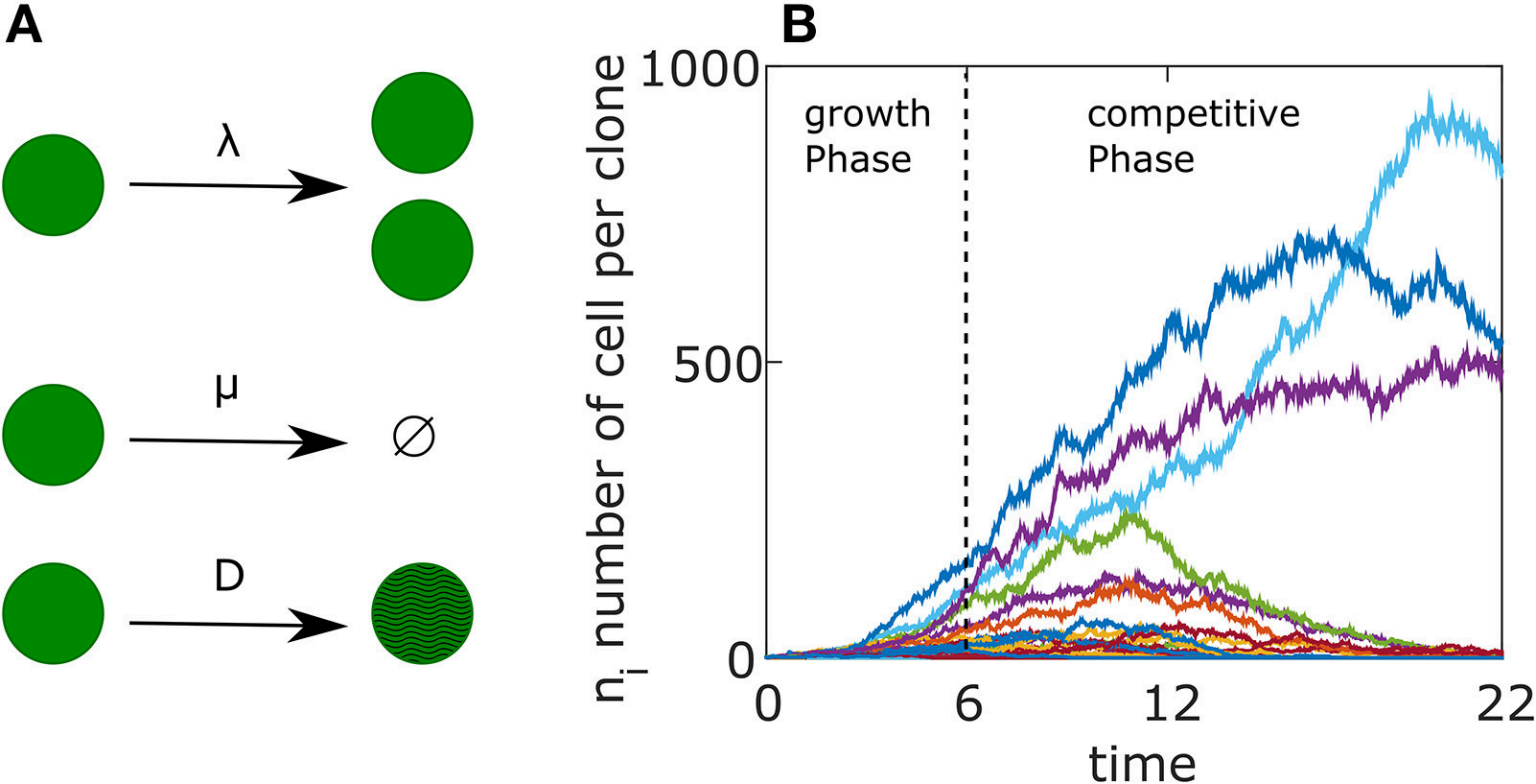
Germinal Center reaction as a birth-death-mutation process. **(A)** Schematics representation of the agent based model. Each cell has a birth rate (λ), a death rate (μ). Upon division the BCR affinity changes according to Equation (9) with a constant *D*. **(B)** Example of a single simulation. The free growth phase lasts for 6 days, followed by a competitive phase lasting 16 days. Each colored curve represents a different clone. The parameters used in the simulation are detailed in Table 1 but with *D* = 0.01.

In the first days following immunization, while the GC is still coalescing, B cells proliferate without competition, creating a pool of cells on which AM may operate. Few or no mutations are introduced to the BCR sequence at this early stage. We start from a simple birth/death (BD) process using an agent-based model. Each cell is associated with a birth rate λ and a death rate μ (see Figure 1A). We assume that a GCR starts with *M* different clones and the system evolves for a period of 6 days, which we denote by *T*_*growth*_ (Jacob et al., 1991; see Figure 1B).

During the growth phase, the probability distribution *P*_*n*_*i*__(*t*) of the number of cells *n*_*i*_ that belong to clone *i* evolves in time according to the master equation (Bailey, 1990):

(1) $$\begin{array}{l} \frac{\partial P_{n_{i}}\left(t\right)}{\partial t}={\text{λ}}_{n-1}P_{n_{i}-1}\left(t\right)+\mu_{n+1}P_{n_{i}+1}\left(t\right)\\ \;-\left({\text{λ}}_{n}+\mu_{n}\right)P_{n_{i}}\left(t\right)\text{ f}or\;n_{i}=1,..,\infty ,\\ \frac{\partial P_{0_{i}}\left(t\right)}{\partial t}=\mu_{1}P_{1_{i}}\left(t\right), \end{array}$$

where (in the absence of interactions): λ_*n*_ = *nλ* and μ_*n*_ = *nμ* and *P*_0_*i*__ is the probability of extinction of clone i. The average number of cells 〈*n*_*i*_〉 in clone *i*, after time *t* is given by (Bailey, 1990).

(2) $$\begin{array}{l} \langle n_{i}(t)\rangle =n_{i}(t=0)e^{(\text{λ}-\mu )t}. \end{array}$$

The time dependent extinction probability of a clone is

(3) $$\begin{array}{l} p_{0_{i}}(t)=\frac{\mu (e^{(\text{λ}-\mu )t}-1)}{\text{λ}e^{(\text{λ}-\mu )t}-\mu }, \end{array}$$

and the size distribution of a clone lineage is

(4) $$\begin{array}{l} p_{n_{i}}(t)=(1-p_{0})(1-\text{λ}p_{0}/\mu ){\left(\text{λ}p_{0}/\mu \right)}^{n_{i}}\text{ for }n_{i}\geq 1. \end{array}$$

Both equations are the solution of Equation (1). After *T*_*growth*_, there is a supply of cells on which AM can work, while some clones disappear. This distribution function is the starting point for the competitive phase of the GCR.

For our parameter choices (see Table 1), which represents a GC development, the average lineage size of a clone at the end of the growth phase (6 days) is 〈*n*_*i*_(6 days)〉 = 20 cells, the total number of surviving cells is 〈*N*(6 days)〉 = 1000 cells, while *p*_0_(6 days) ≈ 2/3 corresponding to an average of 50 × 1/3 ≈ 17 surviving clones. This number is lower than the number of surviving clones in Tas et al. (2016) which was 50–200 but as we are interested in the qualitative behavior of the system, we choose a smaller number to facilitate the numerical calculations.

**Table 1:** Values of the parameters used in the simulation presented in Figure 2A.

**Parameters**	**Value**
Number of initial clones: *M*	50
Basal death rate μ_0_	1 day^−1^
Birth rate λ_0_	1.5 day^−1^
Germinal center capacity *N*	2, 000
Diffusion coefficient *D*	0.001
Initial affinity *w*_0_	1.5
Growth phase *T*_*growth*_	6 days
Competitive phase *T*_*comp*_	16 days

### Competition phase

After day 6, B cells survival depends on TfhC signals that are a shared resource. Indeed, it has been shown (Victora et al., 2010; Gitlin et al., 2015) that TfhCs have a role in regulating the duration of cell cycle in B cells during AM and controlling their behavior in the GC. To mimic B cell competition over the limited resource of TfhCs, we used the stochastic logistic growth process (Nåsell, 2001), which constrains the B cell population size. The death rate decreases with the population size, from a basal rate of μ_0_, to roughly the birth rate λ_0_ for a mature population:

(5) $$\mu (n)=\left(\mu_{0}+\left(\lambda_{0}-\mu_{0}\right)\frac{\sum_{i=1}^{M}n_{i}}{N}\right),$$

where *N* is the population capacity. Here **n** = (*n*_1_, *n*_2_, …, *n*_*M*_) is the vector of cell number *n*_*i*_ for the *M* lineages. The competitive phase continues for a period (*T*_*comp*_), which we take to be 16 days (Tas et al., 2016). The total number of cells in the GC grows gradually until reaching the capacity *N*, where it remains approximately fixed.

### Birth limited selection

Occasionally, B cells undergo a proliferative burst that is proportional to the amount of presented antigen and thus to the BCR affinity (Victora et al., 2010; Gitlin et al., 2015). B cells move then to the DZ, remain there and divide multiple times (4–6) before going back to the LZ to go through another round of selection (Gitlin et al., 2014, 2015; Tas et al., 2016). We model this process as an increase in the birth rate (see Supplementary Information “Heterozygosity of a Moran process”). Since cell-cycle is modified (shortened) in this process, we take the birth rate of cell *i* as

(6) $$\begin{array}{l} \lambda_{i}=\lambda_{0}\frac{w_{i}}{{\langle w\rangle }_{Population}}, \end{array}$$

where *w*_*i*_ is the affinity of cell *i*, 〈*w*〉_*Population*_ is the mean affinity of the population and λ_0_ is the basal birth rate. Indeed, the average birthrate of B cell clones in a GC, was found to be similar (Anderson et al., 2009) in B cell clones with different affinities. The normalization serves to keep the average population birth rate constant at λ_0_. Since the clone birth rate λ_*i*_ is related to the clone affinity *w*_*i*_, we designate this scenario “*birth limited selection*.”.

### Death limited selection

During the GCR, cells with poor affinity do not receive a survival signal from T helper cells because they do not display a sufficient amount of peptide-MHC molecules. Previous studies model this process by noting that the probability of a B cell being able to successfully compete with other B cells that have internalized antigen and receive T cell help, grows monotonically with the affinity of its BCR for antigen (Zhang and Shakhnovich, 2010; Wang et al., 2015), with surviving cells proliferating at approximately the same rate (Batista and Neuberger, 1998). Additionally, it was found (Anderson et al., 2009) that on average, B cell clones with different affinities differ in their death rate, where the low affinity clone dies at a higher rate than ones with intermediate affinity. Such a scenario is considered “*death limited selection*” in our scheme with a death rate μ that depends inversely on the affinity. To study the consequences of such a selection mechanism, we constructed the following model

(7) $$\begin{array}{l} \mu_{i}^{n}=\mu_{i}(w_{i})+(\lambda -{\langle \mu \rangle }_{population})\frac{\sum_{i}n_{i}}{N},\\ \mu_{i}=A\text{ exp}(-\alpha w_{i}), \end{array}$$

where α is a constant, μ_*i*_ is the death rate of a cell with affinity *w*_*i*_ and $\mu_{i}^{n}$ is the GC-size dependent death rate keeping the population size fixed. Thus, higher affinity is related to a lower death rate.

We also examine a model where the birthrate is normalized over the population and as a result, the average of affinity dependent element of death rate, is constant.

(8) $$\begin{array}{l} \mu_{i}^{n}=\mu_{0}\frac{\mu_{i}(w_{i})}{{\langle \mu \rangle }_{population}}+(\lambda -\mu_{0})\frac{\sum_{i}n_{i}}{N}. \end{array}$$

### Affinty change following BCR mutation

During AM B cells mutate their BCR encoding genes. The effect of a single mutation on fitness in models of Wright-Fisher-like selection is often taken to be small (Park and Krug, 2007; Hallatschek, 2011; Goyal et al., 2012; Tas et al., 2016), which allows analytical treatment of the population dynamics as a diffusion problem. In this spirit, we modeled the effect of mutation as a change in the affinity upon cell division, where one of the daughter cells has the parent affinity and for the other daughter:

(9) $$\begin{array}{l} w_{\text{daughter}}=w_{parent}+\text{N}(0,\sqrt{2D}), \end{array}$$

where N is a normal distribution with zero mean and standard deviation of $\sqrt{2D}$, with *D* akin to an effective diffusion coefficient determining the magnitude of affinity change. Within this model, affinity can increase or decrease with equal probability at every division.

## Results

**Figure 2:**
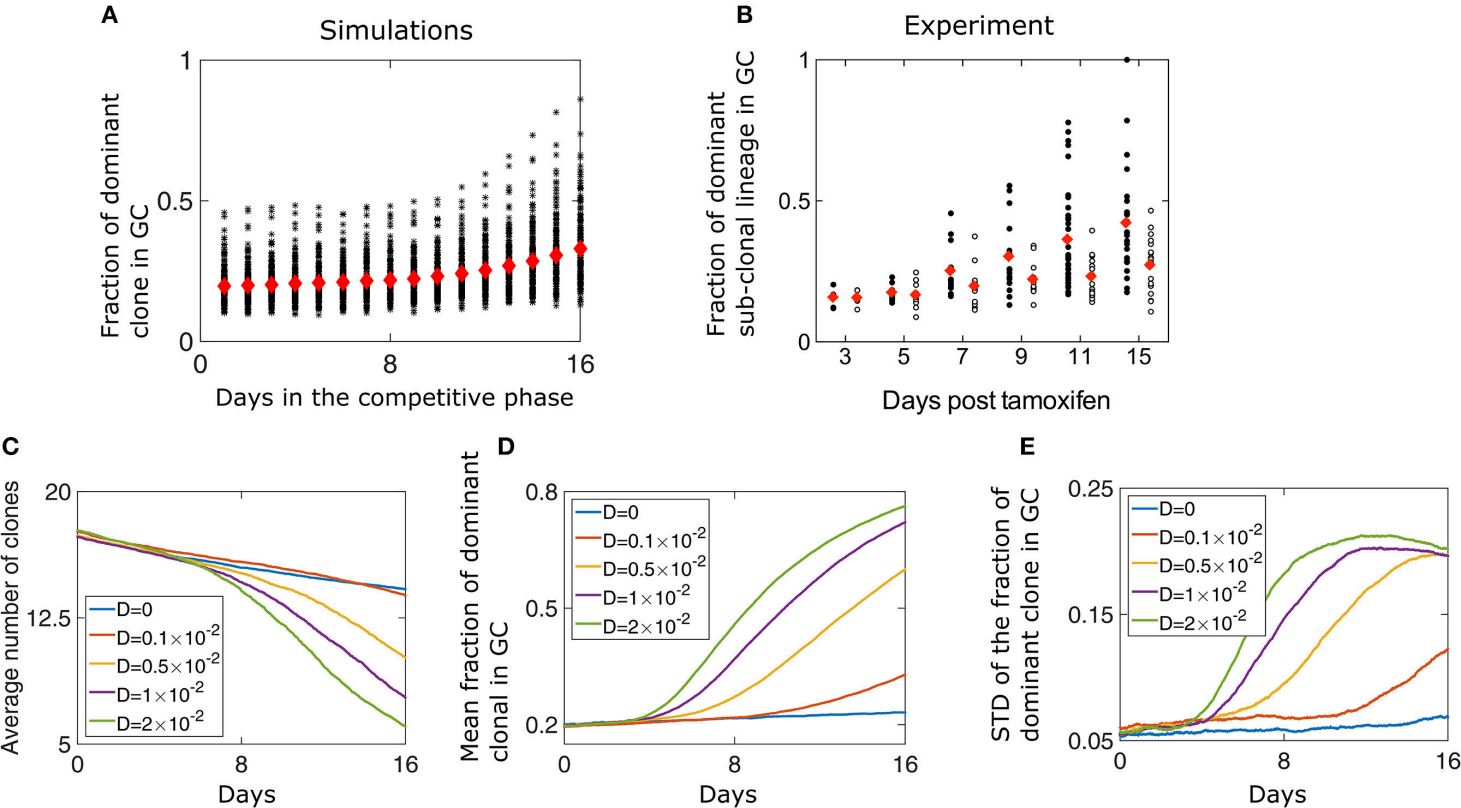
Loss of diversity in a GC. **(A)** The fraction of the GC of size *N* = 2000 occupied by the most dominant clone during the competitive phase. Red diamonds are the mean of 200 independent runs while each black asterisk is the result of a single simulation. The parameters of the simulation are listed in Table 1. **(B)** Fraction of a GC occupied by the dominant sub-clonal lineage, which adopts a unique color upon Tamoxifen-induced recombination (adopted from Tas et al., 2016, Figure 3F). Tamoxifen triggers recombination of one or both Confetti alleles in individual GC B cells, independently of clonal origin. Mice were immunized with chicken gamma globulin at day-5, and GC where B cells participate in the AM process were extracted and analyzed (black circles). Each circle represents one GC. In the control experiment (white circles) all B cells had the same BCR and SHM was prevented by the absence of a functional AID allele. **Clonal size distribution in a GC**. **(C)** Mean number of surviving clones representing loss of clonal diversity during the competitive phase of the GC reaction. The average **(D)** and standard deviation **(E)** of the fraction of the GC of size occupied by the most dominant clone lineage during the competitive phase, for different values of *D*. The simulation started with *M* = 50 at day 0 of the growth phase that lasted 6 days. The parameters used are detailed in Table 1. The results represent 200 independent simulations.

We performed numerical simulations of our model where we started with 50 different clones all having the same initial affinity (*w*_0_ = 1.5) and progressed the reaction in a GC with capacity *N* = 2, 000, which is the characteristic size of GCs in mice (Jacob et al., 1991). We track the fraction of the GC occupied by the different clonal lineages and observe a gradual homogenization of clonal diversity (Figure 2A). We qualitatively compare our results to *in vivo* measurements of clonal diversity, where we track the clones and their respective lineages. In the experiment, each initial clone is colored during the formation of the GC with a specific color by the recombination of the confetti allele. Subsequently, the subclonal lineage has the same color (the details of the experiment are explained in the introduction). Using two-photon microscopy, the size of subclonal lineages formed by the descendants of a cell that is permanently fluorescently labeled is measured (Figure 2B). We observe that with time, fewer clones survive in a GC. Additionally, the fraction of the GC occupied by the most dominant clone has a large variability. A similar behavior is observed experimentally as the fraction of the dominant sub-clonal lineage increase over time. The variability of this fraction across different GCs increases as well (Figure 2B; Tas et al., 2016). By sequencing the BCR region of B cells, the linages of the clones could be reconstructed. From these lineages we estimated the fraction of GC occupied by the dominant clone (Figure S1) and found that it is qualitatively similar to the results obtained with the coloring technique.

### Diversity loss depends on the rate of affinity increase

At the end of the growth phase we are left with 17.2 clones on average, consistent with the stochastic simulations (Figure 2C). At this point, the size of remaining lineages has a large variability according to Equation (4). We find that changing the “diffusion coefficient” *D* has a strong impact on the homogenization rate (Figure 2D). For larger values of *D*, fewer clones survive to be part of a mature GC (Figures 2C,D). The participation ratio, which is the probability that two randomly chosen B cells belong to the same clone, also suggests rapid loss of diversity for large value of *D* (Figure S2). Surprisingly, we find that the variability of different GC realizations increases with time (Figures 2A,E). Naturally, at long times diversity is lost and only a few clones are left, and the variation in the fraction of the most dominant clones decreases (Figure 2E). Thus, the highest number of possible outcomes, in clonal variability, occurs at an intermediate time, which for high values of *D*, happens at day 11 of the competitive phase.

The case of a GCR without mutation was also studied experimentally, in a setting in which multiple clones all having the same BCR seeded the GC and the AID gene was genetically deleted (Tas et al., 2016). Interestingly, even with no changes in affinity, there is a gradual and slow homogenization (Figure 2B, empty circles). To study this scenario, we performed numerical simulations in the absence of mutation (*D* = 0) and saw a gradual take over by the dominant clone (Figures 2C–E), as seen experimentally. As all clones have the same affinity, clonal loss and homogenization in this case is due to random drift (Renshaw, 1991). To gain intuition regarding the selection and fixation process, we recall known results for a case where the population size is fixed, corresponding to a Wright-Fisher process (Bailey, 1990). When affinity differences between the clones are neglected and a starting group of *M* clones all occupy the same fraction of the population size, the mean time to fixation of a single clone is given by τ_fixation_ = 2(*M*−1)log(*M*/*M*−1). With non-uniform initial numbers of clones, the probability of a clone to fix is equal to its initial fraction in the population (Bailey, 1990), which in our model is the probability distribution at the end of the growth phase (Equation 4).

### GC clonal diversity negatively correlates with affinity

**Figure 3:**
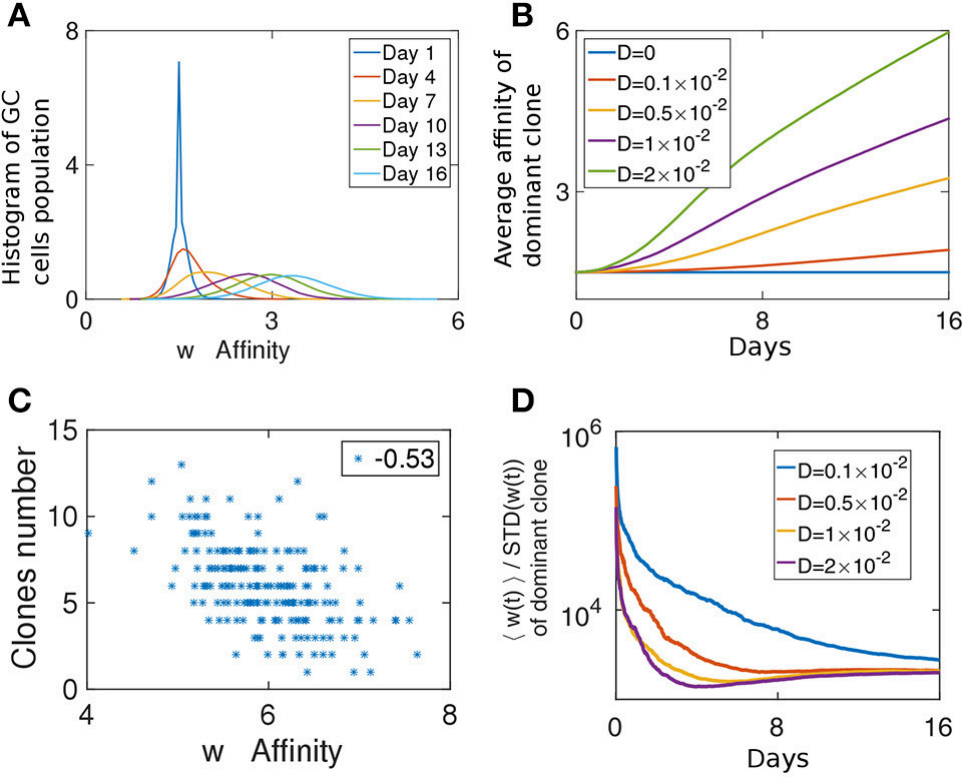
Fitness growth during the competitive phase. **(A)** Affinity distribution of a GC cell population at different days of the competitive phase. Affinity gradually increases as a traveling wave phenomena. The simulation was performed with *D* = 0.005. **(B)** Mean affinity as a function of time for the most dominant clone. Similar parameters were used as in Figures 2C–E. **(C)** Scatter plot of the number of clones in a GC vs. the affinity of the most dominant clone. **(D)** The ratio of the mean affinity of a GC population and its standard deviation.

A clone whose affinity is relatively higher than that of the other clones in the GC has a better chance of being selected and becoming dominant (Equation 6). Since all clones had the same initial affinity, during the first few days of the competitive phase the affinity distribution of the population relaxes from a delta function (δ(*w*−*w*_0_)) (Figure 3A). A GC reaches its capacity only a few days after the beginning of the competitive stage (Figure S3A). Before that, diversity loss continues at the same rate of the growth phase and is *D* independent (Figure 2C). Beyond a certain threshold, the homogenization rate is independent of the birth-rate (Figure S3B).

At later times, the affinity distribution moves as a traveling wave (Tsimring et al., 1996; Hallatschek, 2011; Figure 3A), as fitter strains at the higher end of the affinity distribution function constitute the moving edge while the cells on the other end die. The velocity of the affinity wave depends on *D* (Cohen et al., 2005; Figure 3B) and since affinity changes upon cell division, it depends also on λ (Figure S3D). As expected for a traveling wave solution, the average affinity grows linearly with time. During this period in the GCR, since the affinity of all clones change due to the same stochastic process, a clone which after a single mutation has an affinity larger than the mean, is likely to outperform the other clones. Such deviations from the mean affinity, are governed by large jumps, which are related to the value of *D*.

To study if loss of clonal diversity in a GC is the result of homogenizing selection toward high affinity clones, we computed the correlation between the number of surviving clones in a GC and the average affinity of the most dominant clone at the end of the selection phase (Figure 3C). On day 16, the affinity of the dominant clone is a good proxy for the average affinity in the population. Interestingly, while we observe a weak negative correlation (*r* = −0.53), many GCs maintained diversity in spite of having high affinity clones.

We can consider the width of the affinity distribution of a GC population to be a proxy for its clonal diversity. It was shown that the ratio of the mean affinity to its standard deviation (STD) grows during AM when the amount of antigen used in the immunization was relatively low (Kang et al., 2015). Indeed, the STD of a stochastic variable grows with time (Schuss, 2009), while the growth of the average affinity is evidence of selection (Desai and Fisher, 2007). When the mean grows faster than the STD it is a sign of strong selection. We estimated this ratio from our simulations. Initially, as the affinity distribution spreads from a delta function and before the GC reaches its capacity, the ratio decays, but following the initial relaxation phase, the mean affinity increases faster than the spread of the distribution (Figure 3D). Thus, our system operates in the strong selection limit as in the experimental system studied in Kang et al. (2015).

### Dependence of the final number of cells on the initial growth phase

**Figure 4:**
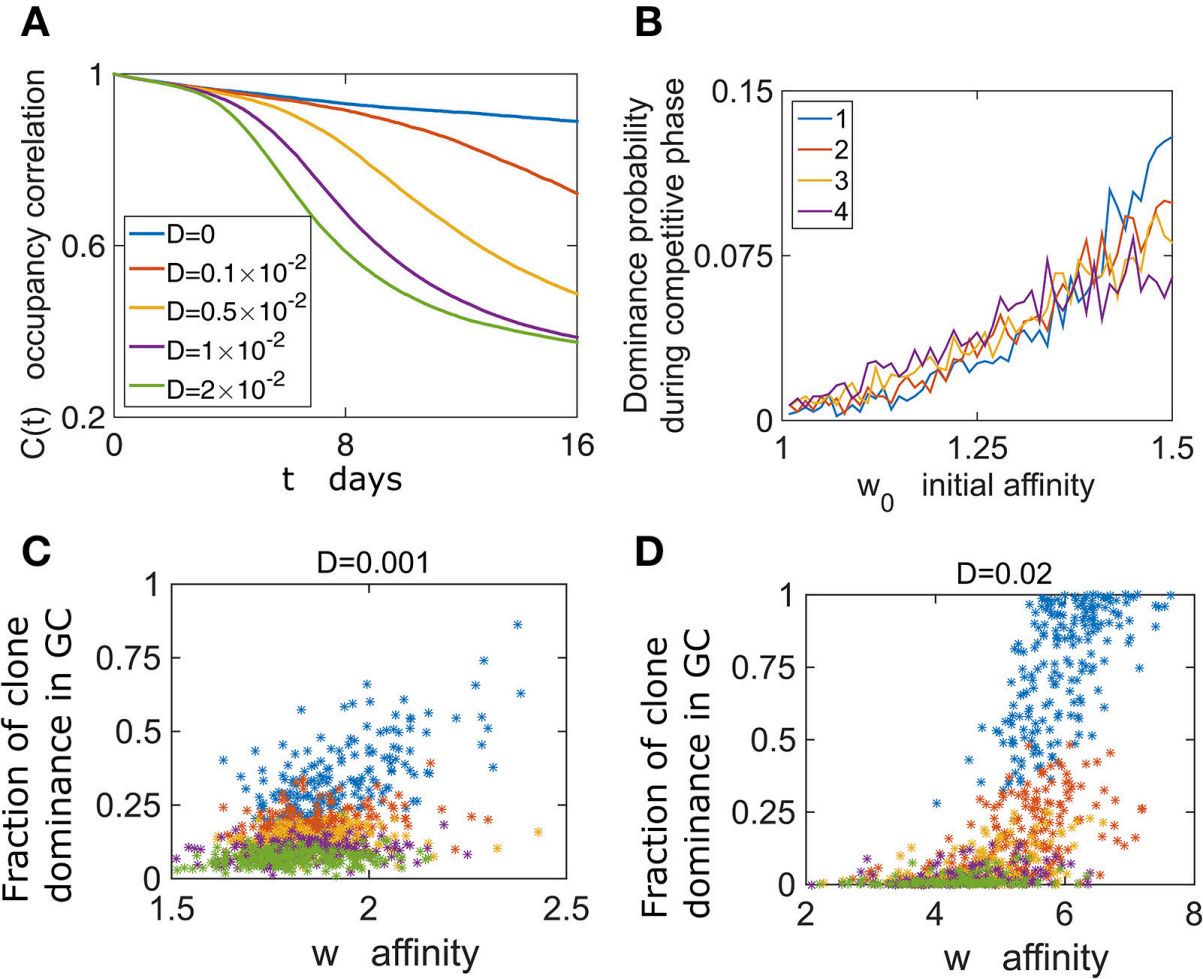
GC content depends on the initial conditions. **(A)** Following growth phase of 6 days, we estimate the occupancy correlation *C*(*t*) Equation (10) during the competitive phase. **(B)** The dominance probability depending on the initial affinity *w*_0_. In the growth phase all cells proliferate with the same rate λ_0_. *w*_0_ determines the birth rate in the competitive phase according to Equation (6) (*D* = 0.02). **(C,D)** The dominance probability is shown for the most dominant clone (blue), second dominance (red), third (yellow) fourth dominance (purple), and fifth (green).

To what extent does the initial growth phase determine the later state of the GCR? We define the state of a GC as the vector of proportions of clonal lineages at time *t*; *n*(*t*) = (*n*_1_(*t*), *n*_2_(*t*), …, *n*_*M*_(*t*))/*N*_*tot*_(*t*). The correlation with the initial state of the GC is quantified by

(10) $$\begin{array}{l} C(t)=\frac{1}{N_{tot}(t)}\sum_{i}n_{i}(T_{growth})n_{i}(T_{growth}+t), \end{array}$$

and is observed to decay with time (Figure 4A). The initial fractions of clones change when stochastic increases or decreases in the affinity of cells give relative advantages or disadvantages to particular clones (Equations 6, 9). Thus, for larger values of *D*, *C* decays faster. Similarly, the decay rate of correlations is inversely proportional to the basal birth rate (Figure S3C) and to *N*, since the fixation probability of a species in a population is inversely proportional to population size (Desai and Fisher, 2007) (data not shown). This result raises the question of whether a GC effectively filters the best clones, as the system has a finite probability to be “stuck” in an unfavorable state.

To further explore the relation between clonal competition and affinity we performed numerical simulations where each B cell of the *M* initial ones had different initial affinity *w*_0_. Following growth, we studied clonal dominance in the competitive phase. Interestingly, while the clone with the highest initial affinity (*w*_0_ = 1.5) had the highest probability of becoming the dominant clone, the clone with *w*_0_ = 1.25 still had a chance of becoming dominant (Figure 4B). This exemplifies the stochastic nature of the selection process. The effect of the initial affinity *w*_0_ in determining the second, third and fourth dominant clone is smaller (Figure 4B).

We addressed the relation between affinity and dominance by estimating the correlation between the average clonal affinity and the fraction occupied by the first to fifth dominant clones. Interestingly, we see that often clones with high affinity compose a small fraction of the GC at the end of the GCR (Figure 4C). We also see that this depends on the value of *D*, and for a larger value the positive correlation between dominance and affinity is stronger (Figure 4D).

### Death limited selection

**Figure 5:**
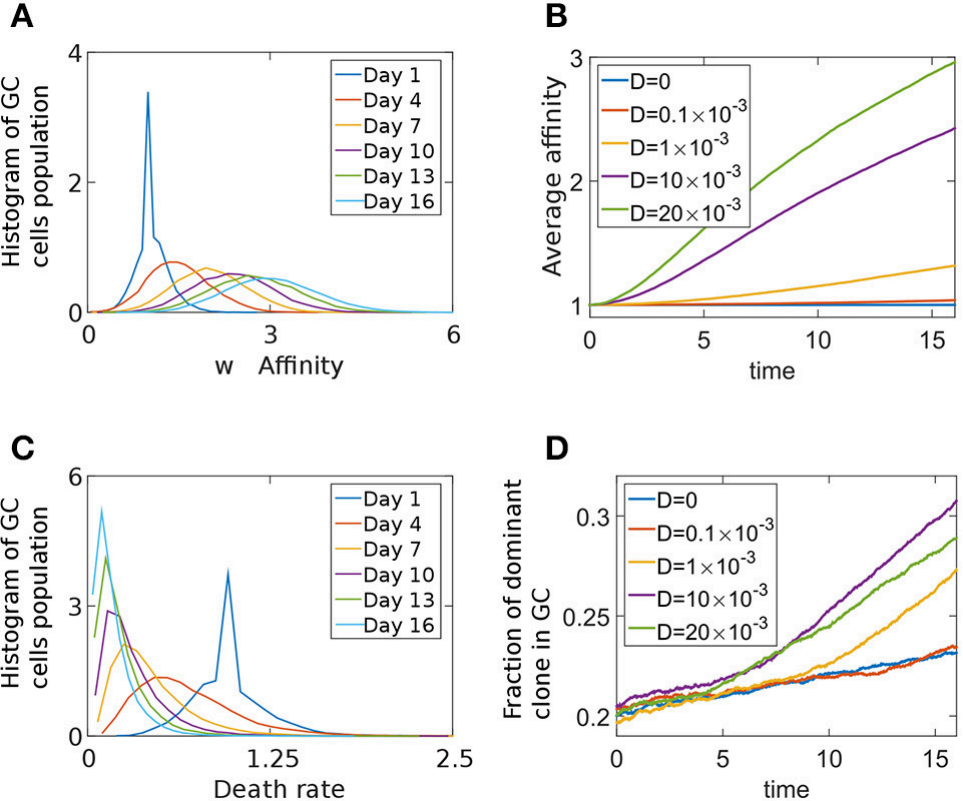
Death limited selection of B cells. **(A)** Affinity distribution of a GC cell population at different times of the competitive phase in the death-limited model Equation (7). The parameters used were: *N* = 2000,*D* = 0.01, λ_0_ = 1.5 day^−1^, α = 1, *A* = *exp*(1) day^−1^, *w*_0_ = 1. **(B)** Average affinity of dominant clone in the death-limited model. **(C)** The death rate distribution corresponding to **(A)**. **(D)** The fraction of the GC occupied by the most dominant clone.

To study the effect of a death-limited model on the progression of the GCR we preform stochastic simulations using an affinity-dependent death rate (Equation 7). The GC population's affinity continues to increase throughout the simulation (Figures 5A,B). We assume that clones with higher affinity have a smaller probability of dying, as they are likely to receive a survival signal from the TfhCs. Thus, in our death-limited model, affinity increase results in decrease of the death rate (Equation 7). Thus, we observe a gradual decrease of the death rate distribution of the cell population (Figure 5C). We found two homogenization regimes (Figure 5D). While the GC has not yet reached its capacity and death rate distribution of the cell population relaxes from a delta function, which was the initial condition (*w*(*t* = 0) = δ(*w* − *w*_0_)), to steady state, homogenization is slow. Indeed, for *D* = 0 the homogenization rate remains constant. In this case, diversity loss is related to random drift only. At later times, homogenization occurs at a fixed rate, dependent on *D* (Figure 5D). The exponential relation between affinity and death rate in this death-limited selection model acts to modulate large affinity jumps. Thus, homogenization occurs at a slower rate than that of the birth-limited model we studied in the previous section.

To investigate if the difference between the death and birth limit selection model is due to normalization of the birth-rate (Equation 6), we performed simulations where the death rate of cell *i* was given by Equation (8). When the average affinity dependent death rate remains μ_0_, the homogenization rate increases (Figure S4) with respect to the un-normalized case, but still remains slower than that for the birth-limited model. There are experimental evidences that the average birthrate is constant in the GC, independent of the affinity of B cells (Anderson et al., 2009). However, such is not the case for death limited selection, since no survival signal is given to B cells by T cells when no Ag is captured. This presumably will occur when the affinity is small. Thus, it is likely that a dependence of death rate and affinity (Equation 7) exists in the GC.

## Discussion

In this study, motivated by recent experimental results, which allowed imaging of AM in GCs over time, we explored simple models to understand the observed phenomenology of clonal selection. The main experimental observation is that clonal selection and homogenization is heterogeneous in a GC population. It appears that the selection of B cell clones, while correlated to the BCR affinity, is probabilistic and lower affinity cells are often selected for proliferation.

We find large variability in the fraction of a GC occupied by different clone lineages. Since selection is a stochastic process, GCs have varying resulting clonal fractions starting from the same founding clone composition. Interestingly, this variability reaches a maximum at intermediate times during the GCR, before decreasing. Our numerical simulations show that the relevant parameter determining homogenization dynamics is the magnitude of affinity modification per single mutation. A large single-mutation change in affinity allows a cell to gain fitness advantage in the population. We find that a fast increase in affinity leads to rapid diversity loss.

Clonal competition can be understood using classical concepts in population dynamics. When the selection pressure is very strong, the fittest variant will survive, that is, the cell with the highest affinity BCR. However, when selection is weaker or when variants compete for different resources, multiple clones or variants can co-exist. The first case is called selective sweep, where one clone dominates over the population (Desai and Fisher, 2007). Alternatively, when selection forces are weaker or mutation rate is fast, clonal interference (Desai and Fisher, 2007) is apparent, where at any time, several clones can coexist. While the first case would result in a relatively homogeneous GC, the second one would appear as a dynamically heterogeneous GC. Interestingly, it appears that both phenomena are possible in different GCs, even ones residing in the same lymph node that have similar initial clonal populations (Tas et al., 2016). This suggests that the GCR lives close to the transition line between the two limiting cases and can stochastically converge in a manner that may depend on the initial conditions, or on fluctuations in the different parameters. We hypothesize that the proliferation boost given to a high affinity (or lucky) B cell can result in a selective sweep. This can presumably occur at any stage of the GC reaction, when a B cell with high affinity manages to capture a lot of Ag and receives multiple proliferation signals from TfhCs leading to multiple divisions in the DZ.

The selection mechanisms we have studied (birth-limited vs. death-limited) result in different homogenization rates and affinities. B cells divide multiple times in the DZ before going back to the LZ. We have shown in the SI that this selection mechanism is equivalent to having a birth rate which is proportional to affinity. This progeny will replace other cells in the GC, thus diversity loss is accelerated. In death-limited selection however, cells with poor affinity are removed one by one. Thus, as a rule, diversity loss in death-limited selection is slower than that of a birth-limited one. For medium and low affinity clones, it was found (Anderson et al., 2009) that they will have approximately the same proliferation rate, while the death depends on the affinity. This could reduce the rate of death-limited selection at later times in the GC, when affinity is higher.

The GCR likely uses these two approaches intermittently. When the fitness landscape of an antibody is very rugged, an optimization algorithm (Bornholdt, 1998) to find a local or global maximum is not effective, as each mutation is likely to greatly decrease the cell fitness. It is possible that the GCR has evolved an approach to use death-limited selection in the LZ as the basal mechanism that would not lead to rapid clonal expansion and GC takeover by a single clone. The second, a birth-rate affinity-dependent selection mechanism, gives a strong proliferation boost to a very successful clone, or to ones that due to random fluctuations managed to capture a large quantity of Ag. Such events may be rarer than death-limited selection, allowing a clone to take over the GC. Thus, diversity is kept as long as no clone distinguishes itself.

We model here selection as a stochastic process using a simple population dynamics model, leading to the gradual homogenization and the variability in GC state. Current experimental results can be recapitulated qualitatively by our coarse-grained model (Figure 2). This suggests that the features we consider are sufficient to recapitulate the qualitative experimental observations regarding diversity loss. Of course, quantitative detailed predictions would require more detailed models including Ag recycling, model of Ag concentration dynamics over time (Tam et al., 2016), explicit description of B-T cells interactions (Meyer-Hermann et al., 2012) can explain the termination of a GCR and interaction between separated GCs in the same lymph node (Figge et al., 2008). Our model could be extended to study complex affinity landscapes and describe AM for multiple antigens and epitopes. It would be interesting to estimate in a high-throughput manner the spectrum of affinities for an antigen and measure the respective selection. Such data could be used to infer the affinity-selection mechanism in a GC.

## Ethics statement

This study was carried out in accordance with the recommendations of, and under protocols approved by, the MIT Committee for Animal Care.

## Author contributions

AA, AC, MK, LM, and GV conceived and designed the *in silico* studies; AA performed *in silico* studies. LM and GV performed experiments. AA, MK, AC, LM, and GV wrote the paper.

### Conflict of interest statement

The authors declare that the research was conducted in the absence of any commercial or financial relationships that could be construed as a potential conflict of interest.

## Supplementary material

The simulation code for this study can be found at: https://amitaiassaf.github.io/
