# Supplementary material for: A Population Dynamics Model for Clonal Diversity in a Germinal Center
Source: Front Microbiol. 2017 Sep 12;8:1693. doi: 10.3389/fmicb.2017.01693 (PMC5600966; doi:10.3389/fmicb.2017.01693)
Supplement: Supplementary file 1 [file DataSheet1.docx]

## Supplementary information

### Heterozygosity of a Moran process

B cells divide multiple times in the DZ, which is proportional to the strength of the metabolic boost they get from TfhCs. The metabolic boost is proportional to the amount of presented antigen and thus to the BCR affinity (Gitlin et al., 2015; Gabriel D Victora et al., 2010). Based on this experimental observation, we built the birth-limited model that we now motivate using a Moran process.

A Moran process (Renshaw, 1991) is an evolutionary model where the population size (*N*) is fixed. Individuals proliferate at a rate 1 and the offspring replaces a randomly chosen individual from the population. To understand the consequence of the multiple divisions before undergoing through a round of selection, we generalize the process to a case where two offspring instead of one are born. Next, two individuals are chosen, each with probability $1/N$ from the current population and are replaced by the offsprings. We assume that there are two alleles in the population. We now compute the heterozygosity which is a measure of diversity of this process. The heterozygosity is the probability than if choose two individuals from a population, they will be of different clones (Renshaw, 1991). For M clones:

$$H=1-\sum_{i=1}^{M} p_{i}^{2},$$

And for 2 clones (alleles) it is given by

$$H(k)=1-\sum_{i=1}^{2} p_{i}^{2}=2\frac{k\left(N-k\right)}{N^{2}},$$

where *k* and (*N-k*) are the number of individuals (cells) of the two clones. The change in time in the heterozygosity is

$$\frac{dH(k)}{dt}=\sum_{j} q_{kj}(H(j)-H(k)),$$

where $q_{kj}$ is the transition rate from having *k* cells of clone one to *j* of them in the population. There are four possible transition rates ($2\leq k\leq N-2$)

$\begin{matrix} q_{k,k+2}=k\frac{\left(N-k\right)\left(N-k-1\right)}{N\left(N-1\right)}, \\ q_{k,k+1}=\frac{k^{2}\left(N-k\right)}{N\left(N-1\right)}, \\ q_{k,k-1}=\frac{k\left(N-k\right)^{2}}{N\left(N-1\right)}, \\ q_{k,k-2}=\left(N-k\right)\frac{k\left(k-1\right)}{N\left(N-1\right)}. \end{matrix}$
For example, consider the rate $q_{k,k+2}$: *k* individuals will prolifirate at a rate of $k$ and the probability of them replacing two cells of type two is $\frac{\left(N-k\right)\left(N-k-1\right)}{N\left(N-1\right)}$. Introducing the rates and the definition of the heterozygosity in eq., we find for $2\leq k\leq N-2$

$$\begin{matrix} \frac{dH(k)}{dt}=\left(q_{k,k+2}H(k+2)+q_{k,k+1}H(k+1)+q_{k,k-1}H(k-1)+q_{k,k-2}H(k-2)\right) \\ -H(k)(q_{k,k+2}+q_{k,k+1}+q_{k,k-1}+q_{k,k-2})=\frac{4k(k-N)(3N-4)}{N^{3}(N-1)}= \\ -H(k)\frac{2(3N-4)}{N(N-1)}. \end{matrix}$$

For large *N* we approximate

$$\frac{dH(k)}{dt}\approx-H(k)\frac{6}{N}.$$

Thus

$$H(t)=H_{0}e^{-t/\tau_{JP}},$$

where $\tau_{JP}=\frac{N}{6}$ is the decay time constant of the heterozygosity in our process. Thus, multiple divisions of a cell before selection, can be capture be a higher birth-rate.

## Supplementary Figures

SI figure 1


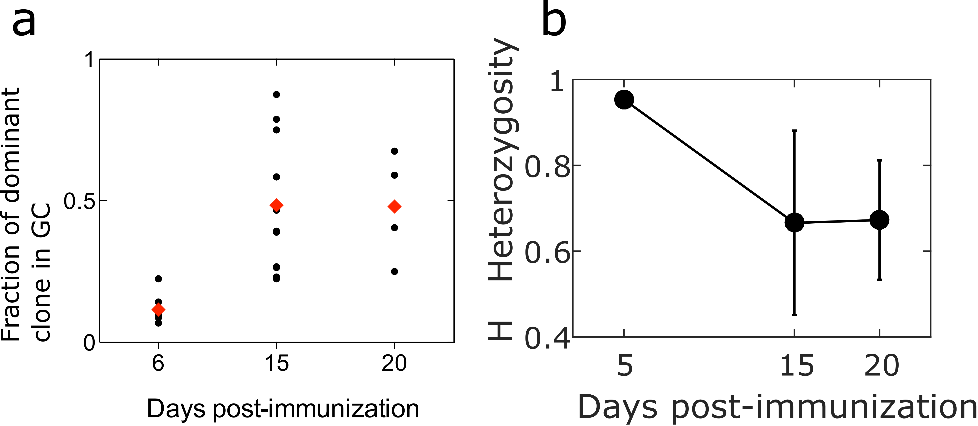


SI figure 2


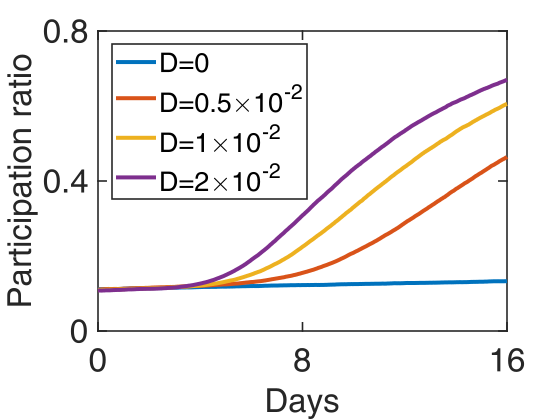


SI figure 3


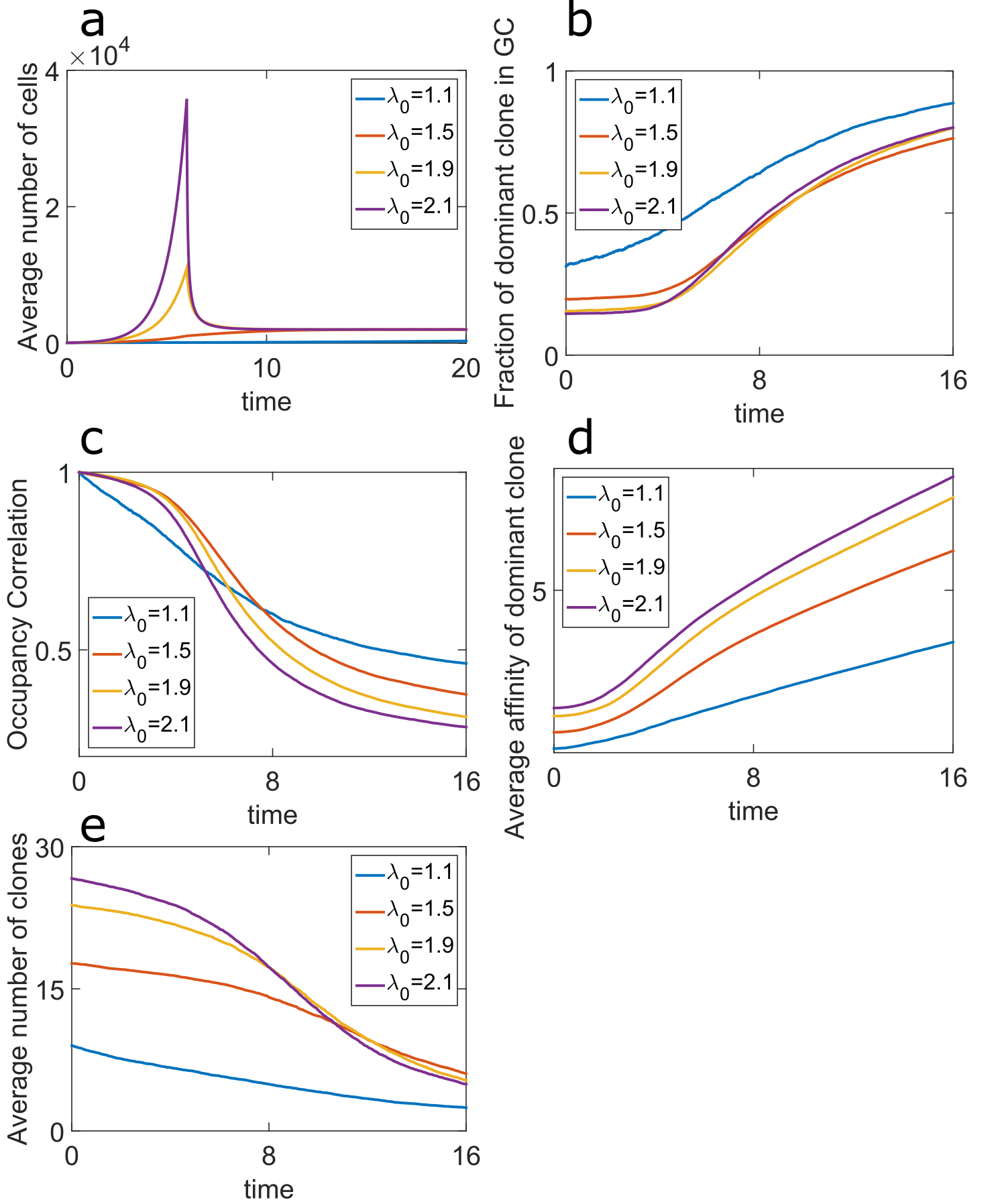


SI figure 4


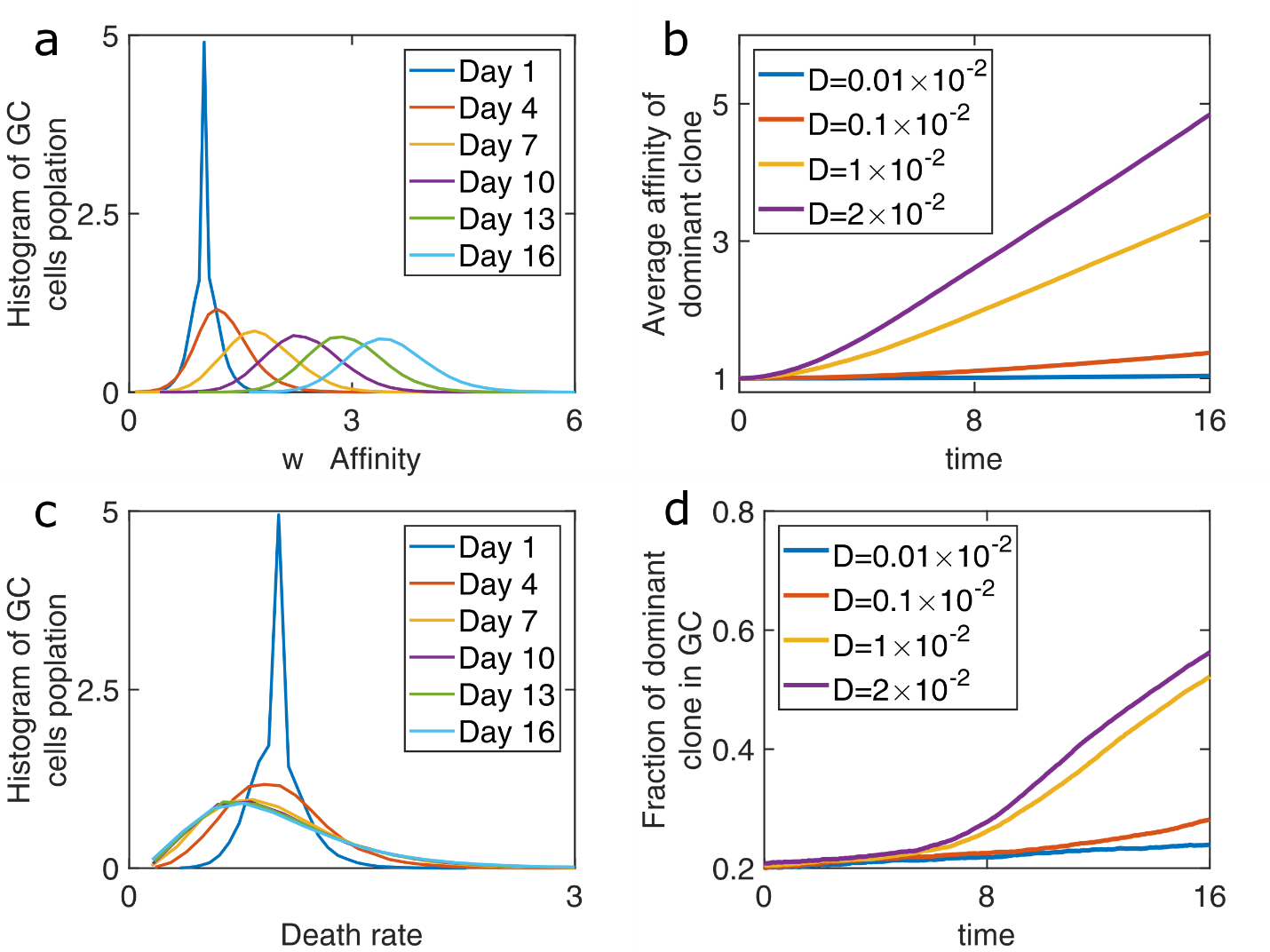


Figure 1 **Clonal diversity in GCs estimated by BCR sequencing**: **(a)** Fraction of the most dominant clone in a GC. Unlike the brainbow system where the relative size of sub-clonal lineages is estimated, here the fraction of a clonal lineage is found by sequencing multiple cell from the same GCs. Sequncing was performed on 8 GCs were sequnced in day 6, 12 GCs in day 15, and 4 GCs in day 20. Data for days 6 and 15 are from (Tas et al); data for day 20 are previously unpublished **(b)** Heterozigocity of the GCs computed for the sequencing experiment using equation . The black dots are the mean heterozygosity among GCs while the error bars are show the standard deviation.

Figure 2 **participation ratio:** The participation ratio of the GC for different value of $D$ averaged over 500 GC realizations. The participation ratio at time t is given by $Y_{t}=\sum_{i} \left(n_{i}(t)/\sum_{j} n_{j}\right)^{2}$(Neher, Vucelja, Mezard, & Shraiman, 2013).

Figure 3: **GCR evolution dependence on division rate.** We study the effect of different birth rates $\lambda_{0}$ on the reaction progression. **(a)** The average number of cells during time $T_{growth}$ (six days) and time $T_{comp}$ (16 days). **(b)** The fraction of GC of size occupied by the most dominant clone during the competitive phase, for different values of birth rates $\lambda_{0}$. Parameters used were: $N=2000$, $M=50,D=0.02,\mu_{0}=1day^{-1}$. **(c)** Following $T_{growth}$, we estimate the occupancy correlation $C(t)$ (eq.) during the competitive phase. **(d)** Mean affinity as a function of time for the most dominant clone. **(e)** Average number of surviving clone representing loss of clonal diversity during the competitive phase of the GC reaction.

Figure 4: **Normalized** **Death limited selection of B cells.** **(a)** Affinity distribution of a GC cell population at different times of the competitive phase in a normalized death-limited model (eq.). The parameters used were the as those used for the simulations in Figure 5. **(b)** Average affinity of dominant clone in the death-limited model. **(c)** The death rate distribution corresponding to (a). **(d)** The fraction of the GC occupied by the most dominant clone. In these simulations the death rate was normalized. Thus the average death rate remains constant.
